# Biochemical and structural investigations clarify the substrate selectivity of the 2-oxoglutarate oxygenase JMJD6

**DOI:** 10.1074/jbc.RA119.008693

**Published:** 2019-05-30

**Authors:** Md. Saiful Islam, Michael A. McDonough, Rasheduzzaman Chowdhury, Joseph Gault, Amjad Khan, Elisabete Pires, Christopher J. Schofield

**Affiliations:** Department of Chemistry, University of Oxford, 12 Mansfield Road, Oxford OX1 3TA, United Kingdom

**Keywords:** RNA splicing, dioxygenase, X-ray crystallography, hypoxia, hydroxylysine (Hyl), hydroxylase, enzyme structure, enzyme catalysis, metalloenzyme, substrate specificity, 2-oxoglutarate and iron dependent dioxygenase, C-5 hydroxylysine, JmjC domain-containing protein 6, JMJD6

## Abstract

JmjC domain-containing protein 6 (JMJD6) is a 2-oxoglutarate (2OG)-dependent oxygenase linked to various cellular processes, including splicing regulation, histone modification, transcriptional pause release, hypoxia sensing, and cancer. JMJD6 is reported to catalyze hydroxylation of lysine residue(s) of histones, the tumor-suppressor protein p53, and splicing regulatory proteins, including u2 small nuclear ribonucleoprotein auxiliary factor 65-kDa subunit (U2AF65). JMJD6 is also reported to catalyze *N*-demethylation of *N*-methylated (both mono- and di-methylated) arginine residues of histones and other proteins, including HSP70 (heat-shock protein 70), estrogen receptor α, and RNA helicase A. Here, we report MS- and NMR-based kinetic assays employing purified JMJD6 and multiple substrate fragment sequences, the results of which support the assignment of purified JMJD6 as a lysyl hydroxylase. By contrast, we did not observe *N*-methyl arginyl *N*-demethylation with purified JMJD6. Biophysical analyses, including crystallographic analyses of JMJD6^Δ344–403^ in complex with iron and 2OG, supported its assignment as a lysyl hydroxylase rather than an *N*-methyl arginyl-demethylase. The screening results supported some, but not all, of the assigned JMJD6 substrates and identified other potential JMJD6 substrates. We envision these results will be useful in cellular and biological work on the substrates and functions of JMJD6 and in the development of selective inhibitors of human 2OG oxygenases.

## Introduction

**Figure 1.**
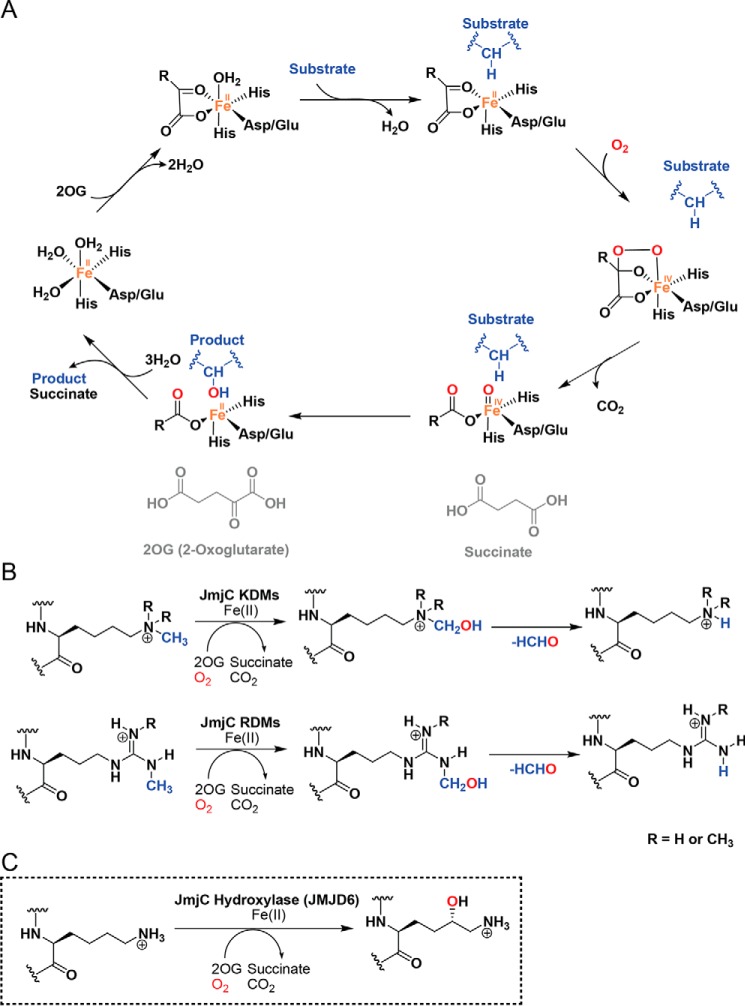
**Schematic of 2OG-catalyzed hydroxylation and demethylation oxidation reactions.** *A,* outline mechanism of a 2OG-dependent oxygenase catalytic cycle; one atom of O_2_ is incorporated into the substrate (R–H), a process coupled to the oxidative decarboxylation of 2OG to give succinate and CO_2_. *B,* reactions catalyzed by JmjC KDMs and JmjC RDMs, *C,* JMJD6-catalyzed lysyl C-5 hydroxylation.

There are ∼60–70 human 2-oxoglutarate (2OG,^3^ α-ketoglutarate) and ferrous iron-dependent oxygenases with diverse biological roles, including in collagen biosynthesis, lipid metabolism, hypoxia sensing, DNA damage repair, and epigenetic regulation (1). Most 2OG oxygenases catalyze two-electron oxidations that are coupled to the conversion of 2OG to succinate and carbon dioxide (Fig. 1*A*) (1, 2). Several human 2OG oxygenases are current clinical drug targets, including the hypoxia-inducible factor prolyl hydroxylases (PHDs) and γ-butyrobetaine hydroxylase (BBOX1) (2, 3).

The JmjC subfamily of 2OG oxygenases is relevant to cancer and other diseases, with functional roles as histone *N*-methyl lysyl and *N*-methyl arginyl demethylases (KDMs and RDMs, respectively) (Fig. 1*B*) (4). JmjC 2OG oxygenase catalyzed *N*-methyl lysyl demethylation proceeds via initial hydroxylation to give a hemiaminal nascent product that normally fragments to give the demethylated product and formaldehyde (Fig. 1*B*) (5). In isolated form, some human JmjC KDMs reportedly manifest a low level of RDM activity (Fig. 1*B*), but the cellular relevance of this remains unclear (4).

A subset of JmjC 2OG oxygenases catalyze the formation of stable alcohol products via the hydroxylation of protein residue side chains (Fig. 1*C*) (1, 6, 7). A prototypical JmjC “hydroxylase” is factor-inhibiting hypoxia-inducible factor (FIH). FIH catalyzes the C-3 hydroxylation of an asparagine residue in the hypoxia-inducible transcription factor (HIF), a modification that serves to regulate the transcriptional activity of HIF, as well as various residues in ankyrin repeat domain proteins (8, 9). Other human 2OG-dependent JmjC “hydroxylases” have been characterized including JMJD5 (an arginyl C-3*R* hydroxylase), JMJD7 (a lysyl C-3*S* hydroxylase), and the ribosomal oxygenases MINA53 and NO66 (both histidine-residue C-3*S* hydroxylases) (1, 6, 10–12). Many of the reactions catalyzed by these JmjC hydroxylases appear to be involved in the regulation of the translation machinery, including via modifications to ribosomally-associated proteins (1, 6, 10–12). Structural differences at the active sites and surrounding regions are proposed to distinguish typical JmjC KDMs and JmjC hydroxylases (7, 10, 13), although given the promiscuity of 2OG oxygenase catalysis, care should be taken in assigning biochemical functions from sequences/structures (1, 2).

JMJD6 is a particularly interesting JmjC family member (14), including from the perspective of its reported enzymatic activities. JMJD6 has been assigned both *N*-methyl arginyl demethylase and lysyl C-5 hydroxylase activities (Fig. 1*C*) (15–17). The protein corresponding to *JMJD6* was initially characterized as the phosphatidylserine receptor (PTDSR) with a consequently associated role in apoptosis (18). Subsequent work, however, established that PTDSR is unlikely to be a membrane protein, instead localizing to the nucleus (19, 20), although it is present elsewhere in the cell (20, 21). Structurally informed bioinformatics led to the prediction that JMJD6 has a JmjC domain containing the modified double-stranded β-helix (DSBH) fold (Fig. S1) that is characteristic of the Fe(II) and 2OG-dependent oxygenases (19, 20, 22). PTDSR was thereafter renamed JMJD6 (19, 23). JMJD6, like FIH (24, 25), contains one domain and forms a homodimer both in solution and in crystals (Fig. S1) (23, 26). Human JMJD6 also has five predicted nuclear localization sequences (Lys^6^–Arg^10^, Lys^91^–Arg^95^, Pro^141^–Lys^145^, Lys^167^–Pro^171^, and Arg^373^–Arg^378^), a predicted AT-hook motif (Lys^283^–Ser^326^), a potential SUMOylation site (Leu^316^–Asp^319^), and a C-terminal polyserine (poly-Ser) region (Ser^340^–Ser^359^, with four interspersed aspartate residues) (19, 20); the JMJD6 poly-Ser region is involved in regulating its oligomerization and cellular localization (Fig. S1) (20, 21).

Chang *et al.* (15) assigned JMJD6 as an histone *N*-methyl arginyl demethylase (RDM) acting on mono- and symmetric/asymmetric di-methyl forms of arginine residues as observed in studies with isolated enzyme (Fig. 1*B*). JMJD6 is also reported to catalyze RDM reactions on *N*-methylated forms of heat-shock protein 70-kDa (HSP70) (27), estrogen receptor α (ERα) (28), RNA helicase A (29), and the stress granule-nucleating protein G3BP1 (GTPase-activating protein SH3-domain–binding protein stress granule assembly factor 1) (30). Although evidence for the interaction of JMJD6 with histones has been observed by several groups, its reported RDM activity is less clear (17, 31, 32).

Subsequent to its assignment as an RDM, JMJD6 was reported to catalyze lysine C-5 hydroxylation (Fig. 1*C*) of the splicing regulatory (SR) proteins luc-7–like 2 (LUC7L2), cisplatin resistance-associated overexpressed protein (CROP), and u2 small nuclear ribonucleoprotein auxiliary factor 65-kDa subunit (U2AF65) (17). JMJD6-catalyzed hydroxylation has been reported to give the lysine C-5–hydroxylated product with the (5*S*)-stereochemistry (Fig. 1*C*) (16), contrasting with lysine residue hydroxylation by the procollagen lysine-hydroxylases which proceeds to give the (5*R*)-product (33). JMJD6 is also reported to undergo self-hydroxylation of lysine residues (34). More recently, the tumor-suppressor protein p53 has been reported to be a JMJD6 hydroxylation substrate (35).

JMJD6 has an apparent role in growth development, with *JMJD6-*knockout mice dying near birth due to growth impairment of multiple organs, including heart, brain, lung, kidney, and eye (36). JMJD6 has been linked to cancer (28, 35), and its hydroxylase activity is linked to preeclampsia via regulation of the HIF system in the placenta (37, 38). However, the details of how these pathophysiological observations are linked to JMJD6 catalytic activity are unclear.

Here, we describe biochemical and biophysical studies on isolated JMJD6 using multiple substrates reported as targets for JMJD6-catalyzed lysine-residue hydroxylation and *N*-methyl arginyl demethylation. The combined results support the assignment of purified JMJD6 as a lysyl C-5 hydroxylase rather than an *N*-methyl arginyl demethylase. This assignment is consistent with crystallographic studies of JMJD6 with its natural ferrous iron cofactor and 2OG cosubstrate; these results imply that the JMJD6 active site is more similar to the JmjC hydroxylases, such as FIH, rather than the JmjC demethylases. We intend that the results will aid in the accurate assignment of JMJD6 substrates in the cellular context and hence help to define its physiological roles.

## Results

Because our objective was to investigate the catalytic reactions catalyzed by isolated JMJD6, the activity of which can be compromised due to oligomerization (14, 31), we initially investigated the catalytic activity of JMJD6 variants: full-length JMJD6 (residues 1–403, JMJD6^FL^), and two C-terminally truncated variants, *i.e.* JMJD6 comprising residues 1–362 (JMJD6^Δ363–403^) and residues 1–343 (JMJD6^Δ344–403^) (Fig. S1*A*). Circular dichroism (CD) spectroscopic analyses imply similar secondary structure content for each of the three JMJD6 variants (Fig. S1*B*).

Hydroxylation assays using the reported LUC7L2_267–278_ substrate (17) revealed moderately increased hydroxylation activity for JMJD6^Δ363–403^ and JMJD6^FL^, both having the poly-Ser region, when compared with JMJD6^Δ344–403^ lacking the poly-Ser region (Fig. S1*C*). The apparent binding constant (*K_D_*^app^) of 2OG was determined using CPMG-edited NMR (39); the *K_D_*^app^ values were 3.5, 3.0, and 2.5 μm for JMJD6^Δ344–403^, JMJD6^Δ363–403^, and JMJD6^FL^, respectively, *i.e.* essentially the same within experimental error (Table 1 and Fig. S2*A*). *K_m_*^app^ values for 2OG were determined using the standard hydroxylation assay procedure and were 31, 24, and 45 μm for JMJD6^FL^, JMJD6^Δ363–403^, and JMJD6^Δ344–403^, respectively (Fig. S2*B*), consistent with Mantri *et al.* (23) who reported a *K_m_*^app^ for 2OG as 39 μm for JMJD6^FL^. NMR monitoring of 2OG turnover in the absence of substrate revealed JMJD6^Δ344–403^, JMJD6^Δ363–403^, and JMJD6^FL^ produce 0.50, 6.4, and 0.90 μm of succinate, respectively, after 1.5 h (Table 1 and Fig. S3). In the presence of the LUC7L2_267–278_ substrate with JMJD6^Δ344–403^, JMJD6^Δ363–403^, and JMJD6^FL^, these values increased to 1.8, 8.4, and 3.6 μm succinate, respectively (Table 1 and Fig. S3). Thus, under both assay conditions, succinate formation for JMJD6^Δ363–403^ and JMJD6^FL^ was higher than JMJD6^Δ344–403^, indicating a possible role of the C-terminal poly-Ser region in enhancing catalysis and/or of active protein stability. However, given the role of the poly-Ser region in oligomerization (21) and the potential for JMJD6 to catalyze hydroxylation of itself (34), the mechanistic interpretation of these results is uncertain. The 2OG turnover assay was also carried out using EDTA-treated JMJD6^Δ363–403^, with freshly prepared Fe(II) solution being added prior to reaction, with the same outcome, *i.e.* with the poly-Ser region containing proteins being more active (Table 1 and Fig. S4). Given that JMJD6^Δ363–403^ was the most active and stable variant tested, further assays were conducted with it.

**Table 1:** **Summary of binding parameters for the cosubstrate 2OG with JMJD6 variants** Succinate formation was monitored in reactions carried out under standard 2OG turnover assay conditions. Values in parentheses are total μm of succinate formed in the 2OG turnover assay using EDTA-treated JMJD6 (with Fe(II) added prior to reaction). *K_D_*^app^ values were determined by following CPMG-edited signals of 2OG titrated against JMJD6. To determine the apparent kinetic parameter (*K_m_*^app^), standard hydroxylation assay conditions were followed with varied concentrations of 2OG (0–150 μm). Values represent mean ± S.D. (*n* = 3).

Variant	Succinate production		2OG	
	Without substrate	With substrate	*K_D_*^app^	*K_m_*^app^
	μ*m*		μ*m*	
JMJD6^FL^	0.90 ± 0.030 (8.0 ± 0.030)	3.6 ± 0.030 (39 ± 0.020)	2.3 ± 1.6	31 ± 5.3
JMJD6^Δ363–403^	6.4 ± 0.020 (12 ± 0.040)	8.4 ± 0.020 (44 ± 0.060)	3.0 ± 4.8	24 ± 5.4
JMJD6^Δ344–403^	0.50 ± 0.020 (1.6 ± 0.050)	1.8 ± 0.030 (5.8 ± 0.030)	3.5 ± 2.1	45 ± 10

### Isolated JMJD6 does not show RDM activity under our assay conditions

**Figure 2.**
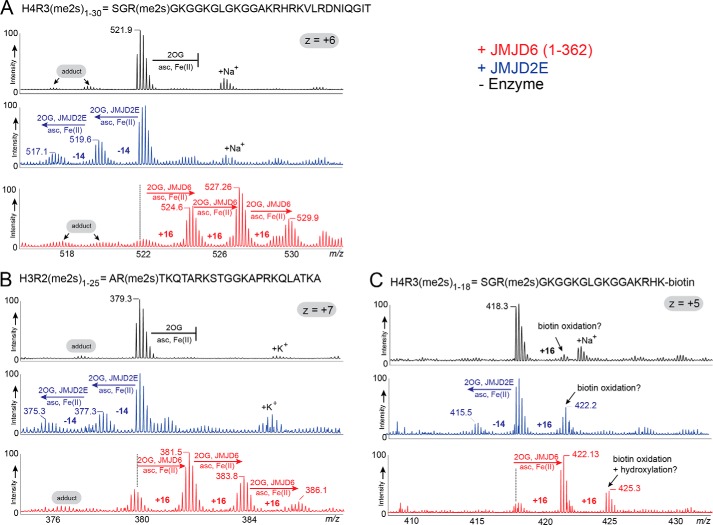
**Evidence that isolated JMJD6 is not a histone *N*-methyl arginyl demethylase.** LC-MS spectra of H4R3(me2s)_1–30_ (*A*), H3R2(me2s)_1–25_ (*B*), and H4R3(me2s)_1–18_-K-biotin (*C*) show the unmodified peptides in the presence of all reaction components except enzyme (*black spectra*). *Red spectra* show peaks with +16-Da mass shifts observed in the presence of JMJD6^Δ363–403^. By contrast, *blue spectra* show peaks with −14- and −28-Da mass shifts for the JmjC KDM JMJD2E/KDM4E-treated peptides suggesting demethylation. Note the lack of evidence for demethylation in the JMJD6-treated substrates. *Adduct*, apparent non-enzymatic peptide modification.

Three histone peptides with symmetrically di-methylated arginine residues, all of which have been reported as JMJD6 RDM substrates (15, 40), were tested using JMJD6^Δ363–403^ under standard assay conditions: H3R2(me2s)_1–25_, AR(me2s)TKQTARKSTGGKAPRKQLATKAA (15); H4R3(me2s)_1–30_, SGR(me2s)GKGGKGLGKGGAKRHRKVLRDNIQGIT (15); and H4R3(me2s)_1–18_, SGR(me2s)GKGGKGLGKGGAKRHK-biotin (40). JMJD2E (KDM4E) was used as a positive control for RDM activity (4). Matrix-assisted laser desorption ionization-TOF (MALDI-TOF) mass spectrometry (MS)-based assays revealed no shift in the masses for peptides in the no-enzyme controls (Fig. 2). Peptides treated by JMJD2E manifested demethylation (peaks with −14 and −28 Da mass shifts) (Fig. 2). By contrast, JMJD6^Δ363–403^ incubations under the same conditions manifested mass shifts of +16, +32, and +48 Da but not of −14 or −28 Da, suggesting JMJD6^Δ363–403^ catalyzes hydroxylation but does not have RDM activity under our assay conditions (Fig. 2). The samples were then analyzed by LC-coupled MS (LC-MS/MS), which showed evidence for demethylation of residues R2_histone3_ and R3_histone4_ in the presence of JMJD2E (Figs. S5–S13). When treated with JMJD6, hydroxylation of K4_histone3_, K9_histone3_, K14_histone3_, K5_histone4_, and K8_histone4_ was observed (Figs. S5–S13), consistent with the MALDI results. Note, we did not accrue any evidence for JMJD6 catalyzed hydroxylation of arginine residues as reported for JMJD5 (12). These results contrast with those of Chang *et al.* (15), who reported JMJD6 RDM activity on H3R2(me2s)_1–25_ and H4R3(me2s)_1–30_ (although their MS results also support hydroxylation).

Evidence for JMJD6^Δ363–403^-catalyzed hydroxylation of lysine residues was also observed with analogous histone peptides H3_1–25_ and H4_1–30_ with unmodified arginine residues (Fig. S14). Furthermore, when a histone peptide monomethylated at Lys^9^ (H3K9me1_1–17_) was tested with JMJD6^Δ363–403^, a +16-Da mass shift was observed, implying hydroxylation and not demethylation (Fig. S15). This observation also suggests JMJD6 does not catalyze histone lysyl demethylation, consistent with an earlier report (17). The level of hydroxylation of Lys^9^(me1) compared with Lys^9^ was relatively low (Fig. 2 and Figs. S14 and S15), which suggests that under our assay conditions JMJD6 prefers to hydroxylate unmodified lysine residues and provides evidence that JMJD6-catalyzed lysyl hydroxylation has the potential to be affected by *N*^ϵ^ lysine modifications.

HSP70 has been reported as a JMJD6 substrate wherein the mono-methylated arginine residue (Arg^469^) is demethylated by JMJD6 as observed by MS (27). However, with JMJD6^Δ363–403^ and a similar peptide to that reported, but without an N-terminal biotin, once again we did not observe demethylation (Fig. S16).

### Steady-state kinetics of JMJD6 catalysis

**Figure 3.**
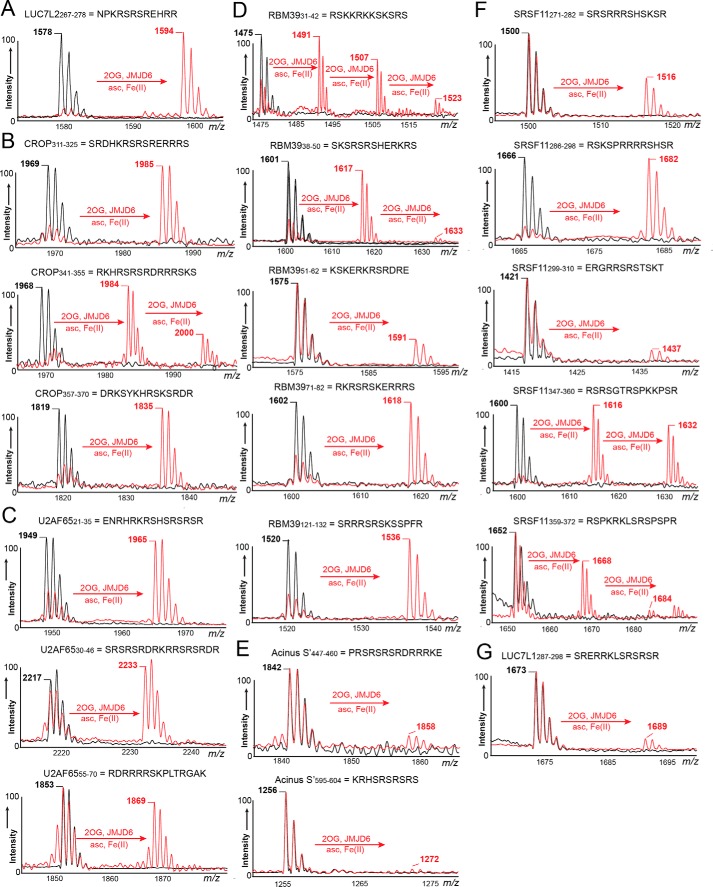
**Evidence that JMJD6 catalyzes hydroxylation of lysine residues from RS-rich regions of SR proteins.** MALDI-TOF MS spectra reveal modifications on JMJD6^Δ363–403^-untreated (*black lines*) and JMJD6^Δ363–403^-treated (*red lines*) peptides from (RS-rich) regions of SR proteins LUC7L2 (*A*), CROP/LUC7L3 (*B*), U2AF65 (*C*), RBM39 (*D*), Acinus S′ (*E*), SRSF11 (*F*), and LUC7L1 (*G*). Experiments were carried out following standard hydroxylation assay procedures (see under “Experimental procedures” for details).

Heim *et al.* (41) have reported JMJD6 interacts with arginine-serine (RS)-rich regions of U2AF65, LUC7L2, SRSF11 (serine/arginine-rich splicing factor 11), and Acinus S′ (apoptotic chromatin condensation inducer in the nucleus), but not with the RS region of SRSF1 (serine/arginine-rich splicing factor 1). Peptides spanning the RS regions of these SR proteins were made and tested as JMJD6^Δ363–403^ substrates, initially screening with fixed time assays and MALDI-TOF MS. The results revealed JMJD6^Δ363–403^-dependent hydroxylation (+16-Da mass shift) (Fig. 3 and Table 2). To investigate whether the observed +16 Da shifts are due to lysyl hydroxylation and the sites of hydroxylation, the lysine residues were systematically replaced by alanine residues. The results enabled assignment of the hydroxylated lysine residues (Figs. S17–S21). Time-course assays were then performed (Fig. S22), with peptides displaying ≥25% hydroxylation after 6 min in kinetic studies (Table 2).

**Table 2:** **Kinetic parameters of the JMJD6-catalyzed hydroxylation reactions of SR protein fragments** Experiments were carried out under standard hydroxylation assay conditions with varied concentrations of the substrate (0–400 μm) (see “Experimental procedures” for details). Values represent mean ± S.D. (*n* = 3).

Peptide	Sequence	pI_calc_ values	Hydroxylation	*K_m_*^app^	*k*_cat_ (× 10^−4^) (s^−1^)	*k*_cat_/*K_m_*^app^ (×10^−6^) (μm^−1^·s^−1^)
			%	μ*m*		
LUC7L2_267–278_	NPKRSRSREHRR	12.18	98.5 ± 4.8	51.0 ± 4.6	106 ± 4.0	208
U2AF65_21–35_	ENRHRKRSHSRSRSR	12.30	74.7 ± 4.1	59.0 ± 8.6	40.0 ± 2.2	68.0
U2AF65_30–46_	SRSRSRDRKRRSRSRDR	12.48	72.4 ± 6.5	78.0 ± 15	92.0 ± 7.0	118
U2AF65_55–70_	RDRRRRSKPLTRGAK	12.30	61.0 ± 6.4	124 ± 34	43.0 ± 5.2	34.0
CROP_311–325_	SRDHKRSRSRERRRS	12.13	89.3 ± 1.9	71.0 ± 7.2	191 ± 6.6	269
CROP_341–355_	RKHRSRSRDRRRSKS	12.40	92.7 ± 6.9	76.0 ± 13	165 ± 12	217
CROP_357–370_	DRKSYKHRSKSRDR	11.00	88.7 ± 4.9	93.0 ± 20	39.0 ± 3.6	42.0
SRSF11_271–282_	SRSRRRSHSKSR	12.60	33.6 ± 1.8	Not determined		
SRSF11_286–298_	RSKSPRRRRSHSR	12.70	90.5 ± 5.0	41.0 ± 6.6	80.0 ± 3.9	195
SRSF11_299–310_	ERGRRSRSTSKT	12.00	6.40 ± 0.90	Not determined		
SRSF11_347–360_	RSRSGTRSPKKPSR	12.48	97.0 ± 2.2	92.0 ± 14	146 ± 9.6	159
SRSF11_358–372_	RSPKRKLSRSPSPR	12.48	44.1 ± 3.9	76.0 ± 21	174 ± 19	229
RBM39_31–42_	RSKKRKKSKSRS	12.32	99.0 ± 0.70	56.0 ± 9.6	255 ± 15	455
RBM39_38–50_	SKSRSRSHERKRS	12.01	79.0 ± 8.9	71.0 ± 9.9	281 ± 15	395
RBM39_51–62_	KSKERKRSRDRE	10.93	37.0 ± 7.6	Not determined		
RBM39_71–82_	RKRSRSKERRRS	12.30	87.6 ± 7.0	88.0 ± 8.7	284 ± 11	322
RBM39_121–132_	SRRRSRSKSPFR	12.60	82.5 ± 5.6	99.0 ± 19	300 ± 23	303
LUC7L1_287–298_	SRERRKLSRSRSR	12.30	18.3 ± 3.1	Not determined		
Acinus S′_447–460_	PRSRSRSRDRRRKE	12.13	25.6 ± 2.8	Not determined		
Acinus S′_95–604_	KRHSRSRSRS	12.48	12.5 ± 3.0	Not determined		

The kinetic parameters obtained for LUC7L2_267–278_ were as follows: *K_m_*^app^, 51.0 μm; *k*_cat_, 106 × 10^−4^ s^−1^; and *k*_cat_*/K_m_*^app^, 208 × 10^−6^ μm^−1^·s^−1^. Of the tested U2AF65 peptide fragments, U2AF65_21–35_ (*K_m_*^app^, 59.0 μm; *k*_cat_*/K_m_*^app^, 68.0 × 10^−6^ μm^−1^·s^−1^) was found to be a better substrate than U2AF65_30–46_ (*K_m_*^app^, 78.0 μm; *k*_cat_*/K_m_*^app^, 118 × 10^−6^ μm^−1^·s^−1^) and U2AF65_55–70_ (*K_m_*^app^, 124 μm; *k*_cat_*/K_m_*^app^, 34.0 × 10^−6^ μm^−1^·s^−1^) (Table 2 and Fig. S23). Other peptides tested, *e.g.* from the SR protein CROP, gave similar kinetic parameters (CROP *K_m_*^app^ value ∼90.0 μm). Of the SRSF11 fragments tested, JMJD6^Δ363–403^ hydroxylated SRSF11_358–372_ with a relatively high efficiency (*K_m_*^app^, 41.0 μm; *k*_cat_*/K_m_*^app^, 195 × 10^−6^ μm^−1^·s^−1^). With RBM39 (RNA-binding protein 39), hydroxylation levels were found to vary with measured *K_m_*^app^ values ranging within 56.0–99.0 μm (Table 2 and Fig. S23). Of the tested RBM39 fragments, RBM39_31–42_ was substantially more efficiently hydroxylated than the others tested, with *K_m_*^app^ 56.0 μm and *k*_cat_*/K_m_*^app^ 455 × 10^−6^ μm^−1^·s^−1^. These results are consistent with the proposal that JMJD6 is promiscuous with respect to its lysyl hydroxylase activity in cells (17, 41); the variations in activities observed imply that more efficient substrates than those studied here are likely to exist within cells.

### JMJD6 hydroxylates lysine residues in pVHL and ERα fragments

The apparent promiscuity of isolated JMJD6 with RS domains of SR proteins prompted us to test the potential of other reported JMJD6 substrates. The von Hippel-Lindau protein (pVHL) has been reported as a potential JMJD6 substrate (37) and was of special interest because of its pivotal role in the hypoxic response via binding hydroxyproline residues in the hypoxia-inducible transcription factors that are produced by 2OG oxygenase catalysis (1, 42). We selected three lysine-containing pVHL sequences for testing with JMJD6^Δ363–403^; MALDI-TOF analysis revealed the presence of a +16-Da mass shift for pVHL_166–177_, but not for pVHL_154–165_ or pVHL_189–200_ (Fig. S24*A*), suggesting possible hydroxylation of Lys^171^. Assays with the alanine variant pVHL_166–177_K171A and with WT pVHL_166–177_ in the presence of a JMJD6 inhibitor, 2,4-pyridine dicarboxylic acid (2,4-PDCA),^4^ did not manifest hydroxylation (Fig. S24*B*). LC-MS/MS analysis of JMJD6^Δ363–403^-treated pVHL_166–177_ provided further evidence for pVHL Lys^171^ hydroxylation (Fig. S24, *C–E*).

Based on Western blot analysis, it has been reported that JMJD6 catalyzes *N*-demethylation of methylated (asymmetrically di-methylated) Arg^260^ of ERα and thereby regulates methylated ERα function in response to estrogen (28). Peptides encompassing residues 251–262 of ERα with symmetrically as well as asymmetrically di-methylated Arg^260^ (MKGGIRKDRR(me2s/a)GG) were tested with JMJD6^Δ363–403^, and ERα demethylation was not observed (Fig. S25*A*). Interestingly, a +16-Da mass shift, corresponding to hydroxylation, was observed for both peptides (Fig. S25*A*). In light of the results with histone peptides, we considered it possible that JMJD6 catalyzes hydroxylation of either or both of the lysine residues (Lys^252^ and Lys^257^) in the ERα sequence. Assays using ERα peptides with unmodified Arg^260^ also revealed JMJD6^Δ363–403^-dependent hydroxylation (Fig. S25*B*). Hydroxylation was ablated by alanine substitution ERα_251–262_K252A, ERα_251–262_K257A, or by addition of the JMJD6 inhibitor 2,4-PDCA^4^ (Fig. S25, *C* and *D*). These results suggest Lys^252^ and Lys^257^ of ERα are possible targets for JMJD6 hydroxylation and are further evidence that JMJD6-catalyzed hydroxylation is possibly regulated by other post-translational modifications.

JMJD6 lysyl hydroxylase activity has also been associated with post-translational modification of the tumor-suppressor protein p53, wherein the target residue is Lys^382^ (35). Assays with JMJD6^Δ363–403^ validated this observation, *i.e.* a +16-Da mass shift of p53 was observed under standard assay conditions implying hydroxylation (Fig. S26).

### Crystallographic studies on JMJD6

**Figure 4.**
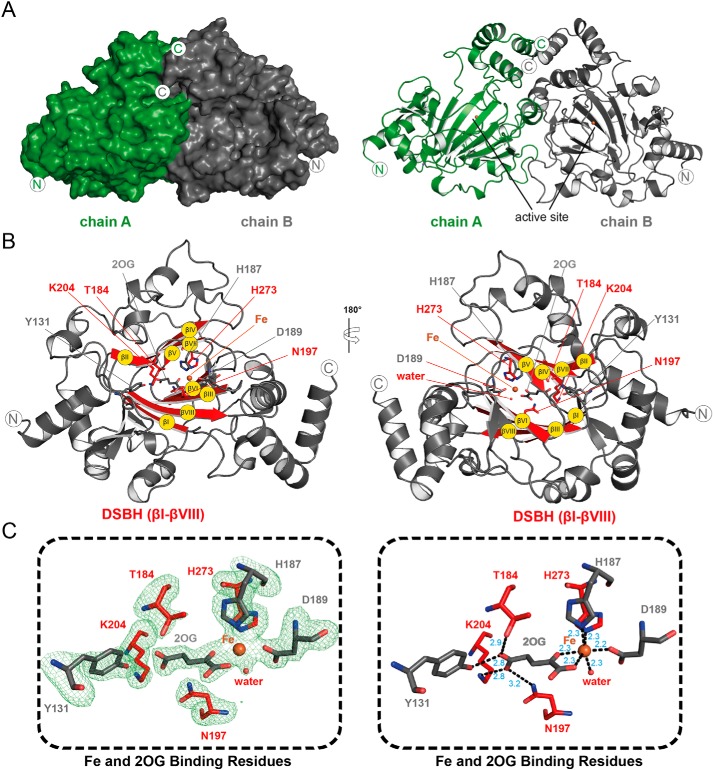
**Crystallographic studies of the JMJD6^Δ344–403^·Fe·2OG complex.** *A, surface* and *cartoon* representations of a crystal structure of the JMJD6^Δ344–403^ homodimer (PDB code 6GDY). *B,* JMJD6 core 8 β-strands of the DSBH fold are *highlighted red* and *labeled* as standard roman numerals βI–βVIII (*yellow circles*); with its cofactor iron shown as an *orange sphere*, and cosubstrate 2OG and active-site residues shown as *sticks. C,* views of the JMJD6 iron- and 2OG-binding residues. The *left panel* shows difference electron density m*F_o_* − D*F_c_* OMIT map (*green mesh*) contoured to 3σ. *Right panel* shows ligand interaction (*black dashes*) distances in angstroms (*blue*).

Crystallization trials were carried out using the three JMJD6 variants. Crystallization of JMJD6^Δ344–403^ in the presence of Fe(II) and 2OG was performed in an anaerobic glove box as reported for FIH (Table 3 and Fig. 4) (24).

**Table 3:** **Crystallographic data collection and refinement statistics**

Protein	JMJD6(1–343)·Fe·2OG (PDB code 6GDY)
X-Ray source	DLS I02
Wavelength (Å)	1.07206
Resolution (Å)	42.22–2.04 (2.11–2.04)
Space group	*P*12_1_1
Unit cell dimensions	
*a, b, c*	47.15, 96.99, 93.76
α, β, λ	90.00, 95.84, 90.00
Molecules/asymmetric unit	2
Wilson *B* factor (A^2^)	32.37
Reflections	53,217 (5258)*^a^*
Completeness	99.6 (99.4)*^a^*
Multiplicity	6.7 (6.6)*^a^*
〈*I*/σ*I*〉	9.7 (1.4)*^a^*
*R*_merge_	0.130 (1.297)*^a^*
CC_½_	0.997 (0.569)*^a^*
*R*_cryst_	0.1795*^b^*
*R*_free_	0.2162*^b^*
Deviation from idealized geometry	
Bond length (Å)	0.003
Bond angles (°)	0.58
Ramachandran plot	
Favored (%)	97.7
Outlier (%)	0.00

*^a^* High resolution bin was used.

*^b^*4.9% of the total reflections used for *R*_free_ calculations is shown.

**Figure 5.**
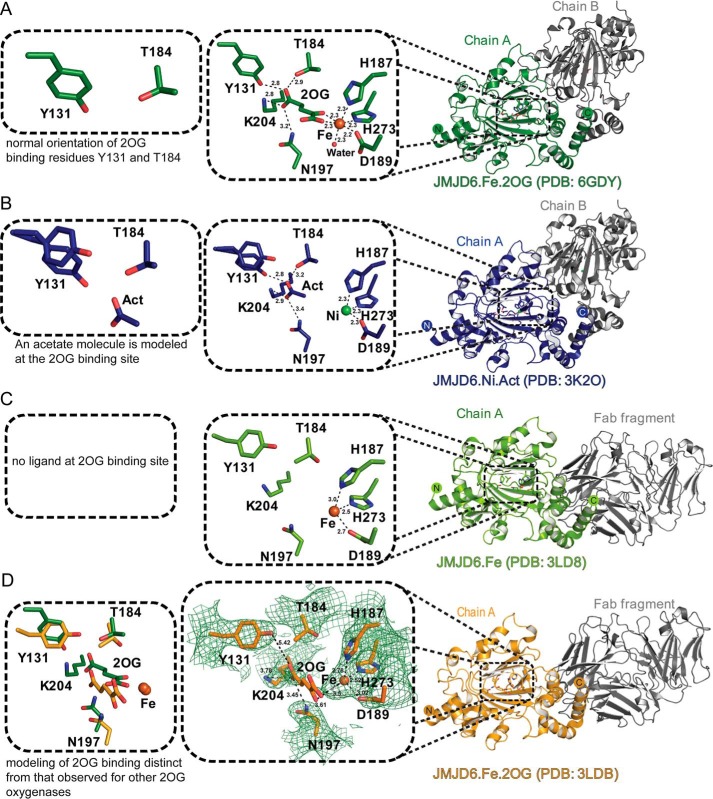
**Comparison of reported crystal structures for JMJD6.** *Cartoon* representations of high resolution crystal structures of anaerobic JMJD6 in complex with iron and 2OG, JMJD6^Δ344–403^·Fe·2OG (PDB code 6GDY) (*A*), compared with aerobic JMJD6 in complex with nickel and acetate, JMJD6^Δ344–403^·Ni·acetate (PDB code 3K2O) (23) (*B*), an aerobic FAB fragment, JMJD6, and Fe complex, JMJD6^FL^·Fe (PDB code 3LD8) (43) (*C*), and low-resolution aerobic FAB fragment, JMJD6, 2OG, and Fe complex, JMJD6^FL^·Fe·2OG (PDB code 3LDB) (43) (*D*) are shown. The *insets* show differences observed in the 2OG/ligand-binding residues in the different crystal structures. *D, left panel* shows a comparison of the active sites from PDB structures 3LDB (*orange*) and 6GDY (*green*). *Middle panel,* note the absence of electron density for 2OG in 3LDB compared with Fig. 4*C, left panel,* showing high resolution structure 6GDY. *Act* = acetate.

The JMJD6^Δ344–403^·Fe·2OG crystals belong to the same space group (*P*2_1_) with similar unit cell dimensions to that reported by Mantri *et al.* (23) (Table S1). The structure was solved by molecular replacement using PDB code 3K2O as a search model (23). JMJD6^Δ344–403^ crystallizes as a homodimer with two molecules in the asymmetric unit (Fig. 4) having a total buried surface area of 2086 Å^2^ (calculated Δ*^i^G* −16.8 kcal·mol^−1^) with no Ramachandran or side-chain conformational isomer outliers (Table 3), consistent with the previously reported structure (total buried surface area of 2050 Å^2^ (calculated Δ*^i^G* −16.6 kcal·mol^−1^)) (23). However, the structure differs from the structure reported by Hong *et al.* (43), where JMJD6 in complex with Fab fragments was observed to crystallize as a monomer (Table S1 and Fig. 5). The combined solution studies on full-length JMJD6 and variants, including JMJD6^Δ344–403^, imply the presence of oligomeric states in solution, including in the dimeric form (21) as observed in our structure, which of the reported structures may be considered most likely to represent a catalytically active form of JMJD6. However, it is also important to note that, as revealed by EM, electrophoresis, and other methods, JMJD6 can also form higher oligomerization states for which there is cellular evidence (21). Indeed, nondenaturing MS analysis with full-length JMJD6 showed masses corresponding to monomeric (48,778 ± 35 Da), dimeric (97,664 ± 36 Da), trimeric (146,795 ± 15 Da), tetrameric (209,745 ± 43 Da), and higher oligomerization states of JMJD6, as observed by EM (21), with the dimer being the major observed species (Fig. S27).

In agreement with previous JMJD6 structures (23, 43), the JMJD6^Δ344–403^·Fe·2OG structure has a distorted DSBH (βI–βVIII) core fold (Fig. 4*B*) characteristic of the 2OG oxygenases, which is located within the JmjC domain and harbours the Fe- and 2OG-binding residues. Importantly, the JMJD6^Δ344–403^·Fe·2OG structure reveals the geometry of the JMJD6 active-site coordination chemistry in the presence of its Fe cofactor and 2OG cosubstrate (Fig. 4). The JMJD6 active site and, in particular, the Fe- and 2OG-binding modes appear to be typical for the JmjC family of 2OG-dependent hydroxylases. A triad of residues, His^187^, Asp^189^, and His^273^, forms the metal-binding motif (Fig. 4*C*), a feature characteristic of most 2OG oxygenases (Asp is sometimes substituted with Glu or Gly) (1). The octahedral coordination of the metal is completed by the oxalyl group of 2OG and a well-defined water molecule (Fig. 4*C*). JMJD6 His^187^ and Asp^189^ are located on the loop linking βII and βIII, whereas His^273^ is located on DSBH βVII (Fig. 4*B*). Mutagenesis studies reveal the importance of the Fe-binding residues in JMJD6 catalysis (Fig. S28*B*). Although the JMJD6^Δ363–404^·D189A variant manifests apparent low-level formation of succinate in the absence of substrate (Fig. S28*C*), succinate formation was not observed in the presence of LUC7L2_267–278_ within the limits of detection (Fig. S28*C*), implying the D189A variant does not support productive hydroxylation catalysis. This provides further evidence that substitution of the metal-binding residues of 2OG oxygenases does not always completely block catalysis; for example, in the case of FIH, it has been reported that substitution of the Fe binding aspartate to a glycine is insufficient to completely ablate hydroxylation activity (25).

**Figure 6.**
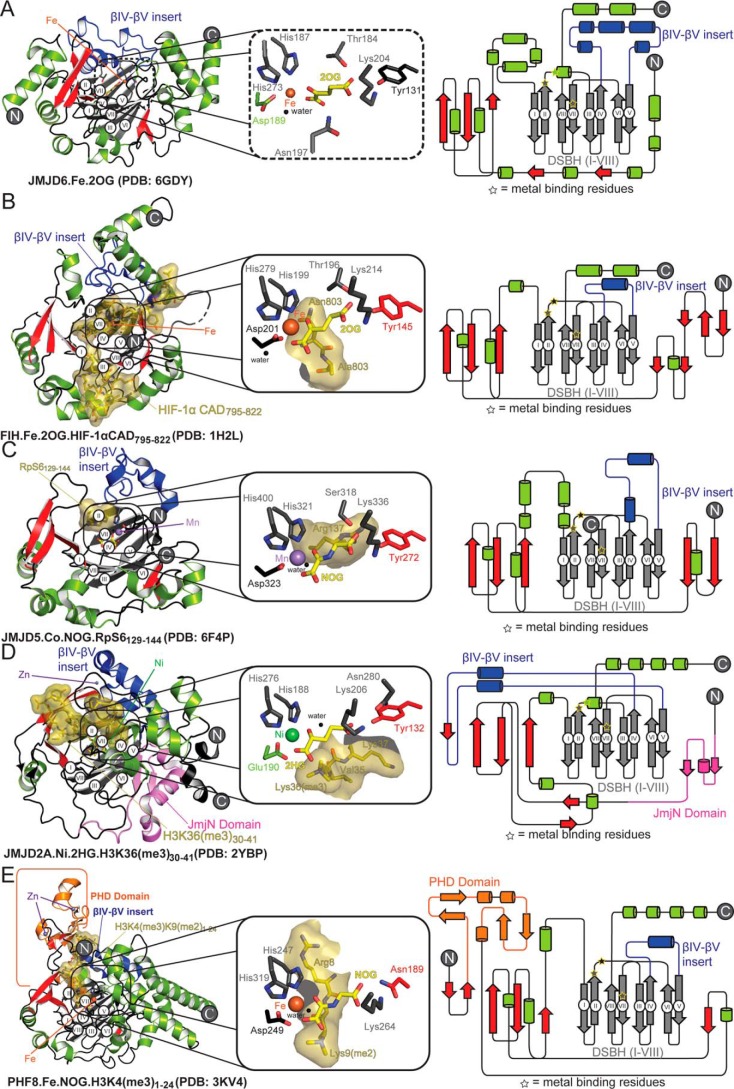
**Comparison of the JMJD6 crystal structure with those of related 2OG oxygenases.** *Cartoon* representations and domain architectures are shown for JMJD6^Δ344–403^·Fe·2OG (PDB code 6GDY) (*A*), compared with JmjC hydroxylases, FIH·Fe·2OG·HIF-1αCAD_795–822_ (PDB code 1H2L) (*B*) (24), JMJD5·Co·NOG·RpS6_129–144_ (PDB code 6F4P) (*C*) (12) and JmjC demethylases, JMJD2A (KDM4A).Ni·2HG·H3K36(me3)_30–41_ (PDB code 2YBP) (*D*) (64), and PHF8(KDM7B)·Fe·NOG·H3K4(me3)_1–24_ (PDB code 3KV4) (*E*) (47). In each enzyme, the core 8 β-strands of the DSBH are in *gray* and labeled (*white circles*), and other β-strands are in *red* unless part of a different domain (*i.e.* PHF8 PHD domain in *orange*). βIV–V insert regions are highlighted with *blue helices*. The insets show an expanded view of the active sites of related enzymes comparing the orientation of substrates (*yellow sticks* and surfaces), metal (*spheres*) and interacting residues (*sticks* colored by region as in cartoon and topology). The locations of metal-binding residues highlighted in topology diagrams (*right panels*) are shown with *yellow stars*.

As predicted, but not observed, by Mantri *et al.* (23), the C-5 carboxylate of 2OG is positioned to form a salt bridge with the side chain of Lys^204^ (βIV) and to make hydrogen-bonding interactions with residues Tyr^131^ (located immediately to the N terminus of βI), Thr^184^ (βII), and Asn^197^ (βIII) (Fig. 4). Similar conformations for Tyr^131^ and Thr^184^ were observed in 3K2O (23), wherein 2OG was absent and where an acetate ion modeled at the active site is positioned to interact with the side chain of Lys^204^ (Fig. 5). Thus, our combined results imply an apparently typical JmjC 2OG-binding mode for JMJD6. By contrast, in the JMJD6·Fab fragment complex structure (PDB code 3LDB), as reported by Hong *et al.* (43), the position of 2OG is different from that observed in other 2OG oxygenase crystal structures, *i.e.* it is relatively distant from the metal and appears to have a poor geometry to form the typical octahedral coordination observed in 2OG oxygenase·Fe·2OG complexes (Fig. 5*D*). Note in the JMJD6^Δ344–403^·Fe·2OG structure, the 2OG C-1 carboxylate is *trans* to the distal His^273^ of the binding triad (His273_JMJD6_) rather than being *trans* to the proximal His^187^ (His187_JMJD6_), as is also observed in FIH, JMJD5, JMJD2A, and PHF8 (*e.g.* His199_FIH_, His321_JMJD5_, His188_JMJD2A_, and His247_PHF8_) structures (Fig. 6). This variation likely does not impact the general mode of iron binding, but it has the potential to direct the binding of the incoming O_2_ *trans* to either the proximal or distal metal-binding histidine depending on the coordination site vacancy. The position of O_2_ binding might play roles in substrate recognition and in regulating the types of reactions that 2OG oxygenases are able to catalyze (2, 13, 44, 45).

**Figure 7.**
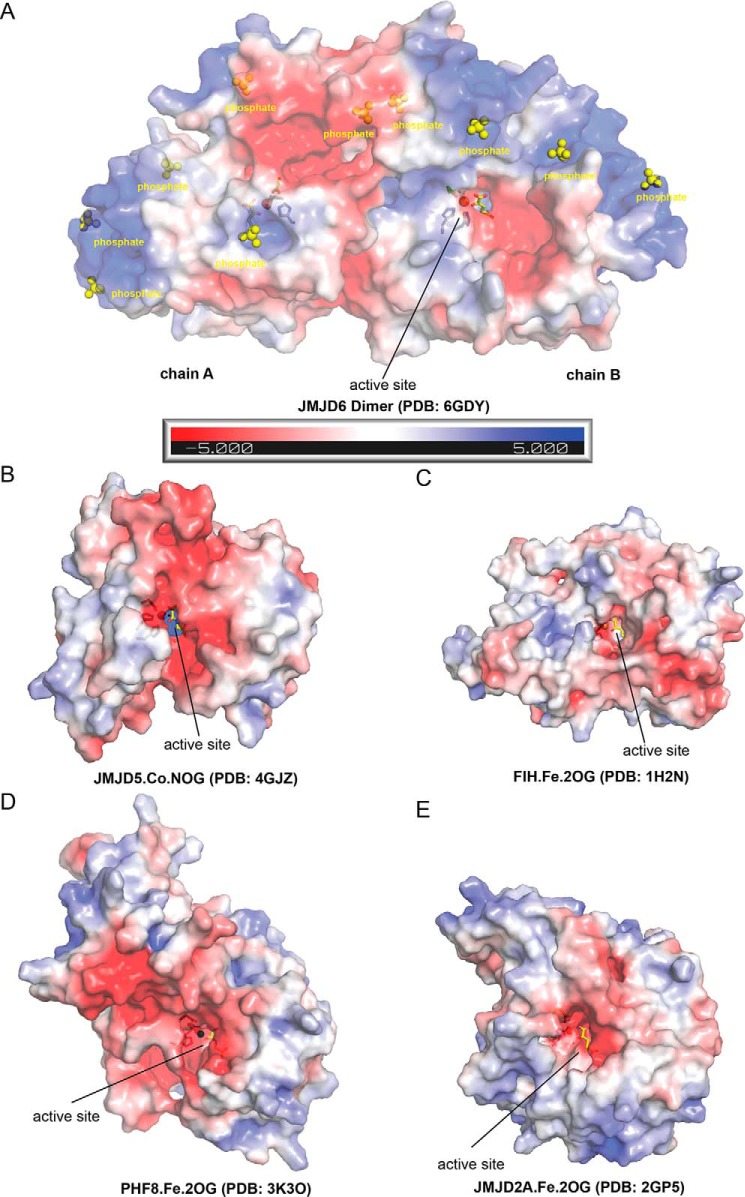
**Comparison of electrostatic surface potential of JMJD6 with those of other 2OG oxygenases.** Electrostatic surface potentials for the crystal structures of JmjC hydroxylases are as follows: *A,* JMJD6^Δ344–403^·Fe·2OG (PDB code 6GDY); *B,* JMJD5·Co·NOG (PDB code 4GJZ) (65); *C,* FIH·Fe·2OG (PDB code 1H2N) (24) and JmjC demethylases; *D,* PHF8(KDM7B)·Fe·2OG (PDB code 3K3O) (66); *E,* JMJD2A(KDM4A)·Fe·2OG (PDB code 2GP5) (67). Note the phosphates (from the crystallization buffer) bound at complementary basic regions may indicate locations of nucleic acid phosphate backbone-binding sites.

Analysis of the electrostatic surface potential (46) of the JMJD6^Δ344–403^ structure reveals that its active site has an overall negative charge (Fig. 7*A*). This is similar to other structurally characterized JmjC domain-containing proteins (Fig. 7, *B–E*) and likely contributes to the ability of JMJD6 to bind the positively charged basic groups of its substrate lysyl side chain as well as other lysyl-/arginyl-residues in the (typically) basic JMJD6 substrates, *e.g.* histones and RS domains of SR proteins.

Following the observation of ordered sulfate molecules at conserved positions across positively-charged sites on the surface of JMJD6 (Fig. S29), Hong *et al.* (43) proposed JMJD6 directly binds oligonucleotides, specifically single-stranded RNA. Consistent with this observation in the JMJD6^Δ344–403^·Fe·2OG structure, we observed binding of phosphate ions in similar locations on the protein surface (Fig. S29), with basic residues involved in these interactions, including Lys^4^, Lys^7^, Arg^8^, Arg^48^, Lys^91^, Arg^92^, Arg^95^, Lys^111^, Lys^115^, Lys^144^, Lys^151^, Lys^154^, Arg^305^, Lys^307^, and Arg^310^ (Fig. S29).

## Discussion

The combined biochemical and structural studies, including those reported here, are fully supportive of the assignment of JMJD6 as a 2OG oxygenase (14). The structure of a JMJD6^Δ344–403^·Fe·2OG complex obtained under anaerobic conditions presented here contrasts with a previously reported structure (43) in that it reveals that the active-site region of JMJD6 is most similar to known hydroxylases and, more specifically the JmjC subfamily, with respect to the observed Fe- and 2OG-binding modes (Fig. 4). The overall negative charge in the active-site substrate-binding region (Fig. 7) is complementary to the primarily basic nature of JMJD6 substrates that have been reported, including histones (15, 32) and ∼RS-rich regions of SR proteins (17). Further analysis shows that the overall architecture of the JMJD6 active site shows greater resemblance to reported JmjC hydroxylases, *i.e.* those giving indefinitely stable alcohol products, than to the JmjC KDMs. JmjC hydroxylases include FIH (24), JMJD5 (12), and JMJD7 (11), all of which form oligomers and manifest a lack of additional substantial discrete domains (Fig. 6). The JMJD6 structural features are more like the JmjC hydroxylases than the KDMs.

Our structural work supports the oligomeric state of JMJD6 as a dimer, in contrast to a monomeric JMJD6 structure reported by Hong *et al.* (43), although a caveat of the latter structure is that it is in complex with a Fab fragment, the binding of which interferes with the dimerization interface. Wolf *et al.* (21) reported the presence of dimeric and higher oligomeric forms of JMJD6 in a cellular context. Most, if not all, JmjC KDMs (and potential RDMs) contain additional domains to the JmjC/DSBH core fold, *e.g.* PHF8 (KDM7B), which has an additional PHD, and the JMJD2 (KDM4) JmjC KDMs (47, 48), which contain JmjN and tudor domains (Fig. 6) (48). However, aspects of the JMJD6 structure, including the role of its characteristic poly-Ser domain in oligomerization (21), appear to be unique among characterized 2OG oxygenases.

As experience shows with 2OG oxygenases, it is imprudent to predict catalytic function based on sequence/structure; even when there is exceptionally high identity, different reactions with closely related enzymes are possible (2, 49); by way of example, one member (TYW5, the transfer ribonucleic acid wybutosine-synthesizing protein 5) of the JmjC subfamily of 2OG oxygenases, which typically acts as protein hydroxylase, has been shown to act on a nucleic acid (50). Thus, our view is that it is important that biochemical studies with isolated enzymes are carried out to define the reactions catalyzed by purified 2OG oxygenases. Although substrate identity/selectivity may well differ in a cellular context compared with that observed for an isolated enzyme, *e.g.* due to the presence of targeting domains/proteins, knowledge of the types of reaction directly catalyzed by the 2OG oxygenase domain *in vitro* can assist in subsequent functional assignment work *in vivo*. Given its checkered history (14), this is arguably particularly important for JMJD6.

Following the identification of an appropriate JMJD6 construct (JMJD6^Δ363–403^), we carried out studies on reported JMJD6 *N*-methyl arginyl “demethylation” and lysyl “hydroxylation” substrates (Figs. 2 and 3, Figs. S14, S22–S26, and Table S2). In our studies, we found no evidence that purified JMJD6 has *N*-methyl arginyl demethylase (RDM) activity (Table S2). Positive controls for RDM activity employing KDM4E (4) were, however, successful (Fig. 2). Unlike the proposed RDM function of JMJD6, the lysyl hydroxylase function of purified JMJD6 was clearly reproducible with a range of substrates, including SR protein fragments (17), histone fragments (15, 17, 32), and the von Hippel-Lindau protein (pVHL) (Table S2) (37). In addition to JMJD6 lysyl hydroxylase activity on peptides, activity was also observed on full-length recombinant and endogenous U2AF65 (17) as well as on endogenous histones (32). Thus, we conclude that, *at least under our assay conditions*, isolated JMJD6 is a lysyl hydroxylase, with no evidence for RDM, nor indeed KDM, activity. Chang *et al.* (15) have reported JMJD6 RDM activity on histones, which they observed using an assay involving the use of antibodies and MS. However, cross-reactivity data for the antibodies used in this study have not been reported. Importantly, the Chang *et al.* (15) MS data also show evidence for JMJD6-catalyzed hydroxylation of histone peptides. It shall also be noted that JMJD6 can also undergo self-oxidation, involving lysyl hydroxylation (34), and that its biological roles may involve stoichiometric as well as catalytic processes (21).

In addition to multiple SR domain substrates (Fig. 3), we found other reports of JMJD6 lysyl hydroxylase activity to be reproducible (Table S2). Lys^382^ of the tumor-suppressor protein p53 is reported to be hydroxylated by JMJD6 (35), and we reproduced this using JMJD6^Δ363–404^ incubated with p53 peptide fragments (Fig. S26). Peptide fragments of two other biochemically important reported targets, *i.e.* pVHL and ERα (28, 37), were also validated as JMJD6 lysyl hydroxylation substrates (Figs. S24 and S25). Although we did not observe JMJD6-catalyzed RDM activity on ERα as was reported (28), hydroxylation of Lys^252^ and Lys^257^ in ERα peptide fragments did occur (Fig. S25).

pVHL is a tumor-suppressor protein in kidneys, which has a crucial role in dioxygen dependent proteasome-mediated degradation of prolyl-hydroxylated forms of α-subunits of the transcription factor HIF in many animal cells (24, 51, 52). In normoxia, HIF is efficiently degraded, whereas proteasomal degradation of HIF-α subunits is slowed because the association between pVHL and HIF1α is diminished due to reduced PHD prolyl hydroxylation activity (24, 51, 52). The results presented here suggest further cell-based studies investigating the potentially pleiotropic roles of JMJD6 lysyl hydroxylation (and potentially other activities) beyond histones and splicing regulatory proteins are merited. Given the established role of pVHL in 2OG oxygenase–mediated hypoxia sensing, its interactions with JMJD6 may be of particular interest.

## Experimental procedures

### Purification of JMJD6

DNA encoding for the N-terminal His_6_-tagged human full-length JMJD6 (aa 1–403, JMJD6^FL^) was inserted into the pET28a(+) expression vector. Expression plasmids for two more variants (aa 1–362, JMJD6^Δ363–403^, and aa 1–343, JMJD6^Δ344–403^) were prepared by inserting a stop codon after the desired site using site-directed mutagenesis (see Table S3 for primer sequences). JMJD6 variants were expressed in *Escherichia coli* Rosetta2(DE3) cells (17, 23). Cells were grown in 2× tryptone/yeast extract (2TY) media supplemented with 30 μg/ml kanamycin and 34 μg/ml chloramphenicol at 37 °C to an *A*_600_ = 1.0; expression was initiated by adding 0.5 mm isopropyl β-d-thiogalactopyranoside. Growth was continued at 16 °C for another 16–18 h; cells were harvested by centrifugation (10,000 × *g*, 8 min) and stored at −80 °C. Cell pellets were resuspended in lysis buffer (50 mm Tris, pH 7.5, 200 mm NaCl, 10 mm imidazole, 10% glycerol, 4 mm MgCl_2_, EDTA-free protease inhibitor mixture tablet (Roche Applied Science), DNase I (bovine pancreas, grade II, Roche Applied Science), and 0.5% Nonidet P-40). The lysates were loaded onto a 5-ml HisTrap column (GE Healthcare), equilibrated with binding buffer (50 mm Tris, pH 7.5, 200 mm NaCl, 10 mm imidazole, 10% glycerol, and 0.5% Nonidet P-40) using an AKTA purifier (GE Healthcare). The column was washed with wash buffer (50 mm Tris, pH 7.5, 200 mm NaCl, 100 mm imidazole, 10% glycerol, and 0.5% Nonidet P-40). JMJD6 was eluted with elution buffer (50 mm Tris, pH 7.5, 200 mm NaCl, 500 mm imidazole, 10% glycerol, and 0.5% Nonidet P-40) using a linear gradient (0–100%). Based on UV-trace (280 nm) and SDS-PAGE analyses, fractions with purified JMJD6 were pooled and concentrated to 2 ml using a membrane filter (30-kDa molecular mass cutoff membrane). Concentrated protein was loaded onto a Superdex 200 size-exclusion column (GE Healthcare), pre-equilibrated with size-exclusion buffer (50 mm Tris, pH 7.5, 200 mm NaCl, and 5% glycerol), for further purification. The desired fractions (based on UV-trace and SDS-PAGE analyses) were concentrated to 25–30 mg/ml, then flash-frozen in liquid nitrogen, and stored at −80 °C. Catalytic mutants of the variant JMJD6^Δ363–403^ were prepared by site-directed mutagenesis (see Table S3 for primer sequences) and were purified by following the same purification procedure as for WT.

### CD spectroscopy

CD spectra were acquired using a Jasco J-815 spectrometer (Jasco). Protein solutions were buffered with 10 mm phosphate buffer, pH 8.0. Spectra were acquired using a quartz cuvette with a 0.1-cm path length, between 260 and 185 nm at 0.2 nm·min^−1^ increments, a response time of 1 s, and a data pitch of 0.5 nm. Spectra were averaged over four scans and corrected for solvent background. To investigate secondary structure content, the CD secondary structure deconvolution method was applied to the data, which were accessed using the DICHROWEB server (53, 54).

### NMR-based 2OG turnover assays

2OG turnover assays employed a reported procedure (4). 10 μm JMJD6, 100 μm (NH_4_)_2_Fe(SO_4_)_2_·6H_2_O (Sigma), and 400 μm l-sodium ascorbate (Sigma) were mixed in deuterated Tris-*d*_11_ (Sigma), pH 7.5, in a 1.5-ml Eppendorf tube. Substrate (either only 500 μm 2OG or a mixture of 500 μm 2OG and 100 μm LUC7L2_267–278_) was added to initiate the reaction. The mixture was immediately transferred into a 2-mm NMR tube, and the acquisition of ^1^H spectra was started after 3.5–5 min.

### NMR-based binding constant (K*_D_*^app^) determination for 2OG

20 μm 2OG and 200 μm Zn(II) were mixed in deuterated Tris-*d*_11_, pH 7.5, and the ^1^H NMR signals for 2OG were recorded at 700 MHz (39). Titration using EDTA-treated JMJD6 (with added Zn(II)) was performed until the signals for 2OG disappeared, suggesting JMJD6·Zn(II)·2OG complex formation. The percentage of this complex was then plotted as a function of enzyme concentration using Origin (OriginLab Corp.), and the apparent binding constants (*K_D_*^app^) were measured using Equation 1 (55),

(Eq. 1) $$\Delta_{\text{obs}}=\Delta_{\text{total}}\times \frac{\left({K_{D}}^{\text{app}}+{\left[L\right]}_{0}+{\left[P\right]}_{0}\right)-\sqrt{\left\{{\left({K_{D}}^{\text{app}}+{\left[\text{L}\right]}_{0}+{\left[\text{P}\right]}_{0}\right)}^{2}-4\times {\left[\text{L}\right]}_{0}\times {\left[\text{P}\right]}_{0}\right\}}}{2\times {\left[\text{P}\right]}_{0}}$$

where Δ_obs_ represents change in an observable NMR parameter from the titration; Δ_total_ represents the total change of the monitored NMR parameter; [L]_0_ represents the titrated ligand concentration; and [P]_0_ represents the protein concentration. Typically, a 5–10% error was allowed in the curve-fitting process.

### NMR-based CPMG displacement experiments

CPMG displacement experiments used a Bruker AV700 NMR machine, as reported previously (39). The PROJECT (Periodic Refocusing of J Evolution by Coherence Transfer)-CPMG sequence (90°*_x_* − (τ − 180°*_y_* − τ − 90°*_y_* − τ − 180°*_y_* − τ)*_n_* − acquisition) was applied (56). Typical parameters were as follows: total echo time 48 ms (τ = 2 ms, *n* = 6); acquisition time 2.94 s; relaxation delay 2 s; number of transients 128. Water suppression was achieved by presaturation (57, 58).

### Standard MS-based hydroxylation assays

JMJD6 activity was assayed using a reaction mixture of 10 μm purified JMJD6 and 100 μm substrate peptide, at 37 °C in 50 mm HEPES-KOH, pH 8.0, buffer supplemented with 100 μm (NH_4_)_2_Fe(SO_4_)_2_·6H_2_O (Sigma), 400 μm l-sodium ascorbate (Sigma), and 500 μm disodium 2OG (Fluka). After 30 min, the reaction was quenched by an equal volume of 1% (v/v) aqueous formic acid (Sigma). In a 96-well MALDI plate (Waters), 0.8 μl of CHCA (α-cyano-4-hydroxycinnamic acid, Aldrich) (1 μg in 0.1 ml of CHCA solvent) and 0.6 μl of the quenched mixture were placed in a well. After drying, the samples were analyzed by MALDI-TOF MS using a reported procedure (59, 60). The kinetic data obtained thereafter were fitted using GraphPad Prism (GraphPad Software) (59, 60).

### Standard MS-based demethylation assays

Demethylation assays were carried out using JMJD6 and potential substrate peptides at 37 °C in 50 mm HEPES-KOH, pH 8.0 buffer, supplemented with 20 μm (NH_4_)_2_Fe(SO_4_)_2_·6H_2_O (Sigma), 500 μm l-sodium ascorbate (Sigma), and 1 mm di-sodium salt of 2OG (Fluka) for 1 h or more (15, 27, 40). Reaction samples were thereafter analyzed by MALDI-TOF or LC-MS/MS.

### LC-MS/MS analyses

Samples were desalted using a ZipTip (Millipore) and then analyzed using a NanoAcquity-ultraperformance liquid chromatography system (Waters) connected to an Orbitrap Elite mass spectrometer (Thermo Fisher Scientific), possessing an EASY-Spray nano-electrospray ion source (Thermo Fisher Scientific). Peptides were “trapped” on an in-house packed guard column (75-μm inner diameter × 20 mm, Acclaim PepMap® C18, 3 μm, 100 Å) using solvent A (0.1% (v/v) aqueous formic acid) at a pressure of 140 bar. Peptides were separated on an EASY-Spray Acclaim PepMap® analytical column (75 μm inner diameter × 15 mm, rapid separation liquid chromatography C18, 3 μm, 100 Å) using a linear gradient (length, 100 min; 3–60% solvent B (0.1% formic acid in acetonitrile), flow rate, 300 nl/min). The separated peptides were electro-sprayed directly into the spectrometer operating in a data-dependent mode using a collision-induced dissociation/electron transfer dissociation (CID/ETD)-based method. Full scan MS spectra (scan range 150–2000 *m*/*z*, resolution 30,000, AGC target 1 × 10^6^, maximum injection time 250 ms) and subsequent CID MS/MS spectra (AGC target 3 × 10^4^, maximum injection time 500 ms) of the 10 most intense peaks were acquired in the Orbitrap. CID fragmentation was performed at 35% of the normalized collision energy, and the signal intensity threshold was kept at 500 counts. ETD spectra (AGC cation target 5 × 10^4^, AGC anion target 2 × 10^5^, and cation maximum injection time 100 ms) were acquired in the ion trap. PEAKS® 8.0 (Bioinformatics Solutions, Inc., Waterloo, Canada) was used to analyze raw data. The raw MS files were searched against the respective protein sequences. NONE was selected as the protease. Demethylation (−14 and −28 Da) and hydroxylation (+16, +32, and +48 Da) were set as variable modifications. The precursor mass tolerance was 15 ppm. The fragment mass tolerances for CID and ETD were set to 0.8 Da. All spectral assignments were manually validated.

### Crystallization and structure determination of JMJD6^Δ344–403^

Crystallization trials were performed using commercially available broad screens followed by optimization. JMJD6^Δ344–403^ of high purity (≥95%, based on SDS-PAGE analysis) at a concentration of ≥25 mg/ml (500–600 μm) was used. JMJD6^Δ344–403^·metal·ligand complexes were formed by incubating enzyme with the other components (typically in 5-fold excess) on ice for ∼1 h. A Phoenix^TM^ RE crystallization robot (Art Robbins Instruments) and a Minstrel-HT^TM^ platform with CrystalTrek^TM^ (Rigaku Inc., Japan) were used to prepare and monitor crystallization trials. Crystals were grown using the sitting-drop vapor-diffusion method (61) either in 96-well low-profile intelliplates (Art Robbins Instruments) (drop size, 200–300 nl) at room temperature or in 24-well Linbro sitting-drop plates (drop size 1000 nl) in an anaerobic glovebox (Belle Technologies, Dorset, UK) to avoid turnover during crystallization as reported previously (24). The optimized crystallization condition contained 0.1 m HEPES buffer, pH 7.8, 0.7 m sodium phosphate monobasic, 0.9 m potassium phosphate monobasic, and 3% glycerol. This crystallization condition was obtained from optimization of an initial hit from a broad screen (Hampton Research Crystal Screen HT, no. 35). Crystals were cryo-protected using a mixture of reservoir solution diluted to 25% (v/v) glycerol and cryo-cooled by plunging into liquid nitrogen. Diffraction data were collected at 100 K at the Diamond Light Source synchrotron. PHASER was used for molecular replacement using PDB code 3K2O (23) as the search model; the structural model was improved by iterative cycles of manual re-building in COOT and crystallographic refinement in PHENIX (Python-based Hierarchical Environment for Integrated Xtallography) (62, 63).

### Data availability

Coordinates and structure factors for the JMJD6^Δ344–403^·Fe·2OG structure are deposited in the Protein Data Bank as code 6GDY.

## Author contributions

M. S. I., M. A. M., R. C., and C. J. S. conceptualization; M. S. I. and M. A. M. data curation; M. S. I. and M. A. M. software; M. S. I. and M. A. M. formal analysis; M. S. I. and C. J. S. funding acquisition; M. S. I. and M. A. M. validation; M. S. I., J. G., A. K., and E. P. investigation; M. S. I. and M. A. M. visualization; M. S. I. methodology; M. S. I., M. A. M., and C. J. S. writing-original draft; M. A. M., R. C., and C. J. S. supervision; M. A. M. and C. J. S. project administration; M. S. I., M. A. M. and C. J. S. writing-review and editing; C. J. S. resources.

## Supplementary Material

Supporting Information

## Footnotes

4 Md. Saiful Islam, unpublished data.

## Footnotes

3 The abbreviations used are:

2OG: 2-oxoglutarate/α-ketoglutarate
2HG: 2-hydroxyglutarate, 2,4-PDCA, 2,4-pyridine-dicarboxylic acid
Acinus S′: apoptotic chromatin condensation inducer in the nucleus
AGC: automatic gain control
CAD: C-terminal transcriptional activation region of HIF-1α
CHCA: α-cyano-4-hydroxycinnamic acid
CID: collision-induced dissociation
CPMG: Carr-Purcell-Meiboom-Gill
CROP: cisplatin resistance-associated overexpressed protein
DSBH: double-stranded β-helix
ERα: estrogen receptor α
ETD: electron transfer dissociation
FAB: fragment antigen binding
FIH: factor-inhibiting hypoxia–inducible factor
H3: histone 3
H4: histone 4
H4R3(me2s): histone 4 arginine 3 symmetrically di-methylated
HIF: hypoxia-inducible transcription factor
JmjC: Jumonji C
JMJD: JmjC domain-containing protein
KDM: *N*-methyl lysyl demethylase
KDM4E: lysine-specific demethylase 4E
KDM7B: lysine-specific demethylase 7B
LUC7L: luc-7–like
NOG: *N*-oxalylglycine
PDB: Protein Data Bank
PHD: prolyl hydroxylase
PHF8: plant homeobox domain (PHD) finger protein 8
PTDSR: phosphatidylserine receptor
pVHL: von Hippel-Lindau protein
RDM: *N*-methyl arginyl demethylase
RS: arginine-serine
SR: splicing regulatory
SRSF11: serine/arginine-rich splicing factor 11
aa: amino acid.
