# Supplementary material for: Biochemical and structural investigations clarify the substrate selectivity of the 2-oxoglutarate oxygenase JMJD6
Source: J Biol Chem. 2019 May 30;294(30):11637–52. doi: 10.1074/jbc.RA119.008693 (PMC6663879; doi:10.1074/jbc.RA119.008693)
Supplement: Supporting Information [file supp_294_30_11637__index.html]

Biochemical and structural investigations clarify the substrate selectivity of the 2-oxoglutarate oxygenase JMJD6 — Substrate selectivity studies of the 2OG oxygenase JMJD6 — Supporting Information 

# Biochemical and structural investigations clarify the substrate selectivity of the 2-oxoglutarate oxygenase JMJD6

## Supporting Information

- Supporting Information (to be published online) - PDF file of the Supporting Information
