# Supplementary material for: Biochemical and structural investigations clarify the substrate selectivity of the 2-oxoglutarate oxygenase JMJD6
Source: J Biol Chem. 2019 May 30;294(30):11637–52. doi: 10.1074/jbc.RA119.008693 (PMC6663879; doi:10.1074/jbc.RA119.008693)
Supplement: Supporting Information [file supp_RA119.008693_144648_1_supp_333565_prp6kq.pdf]

The neuropeptide GsMTx4 inhibits a mechanosensitive BK channel through the specific modulation of a mechano-sensing gate depending on membrane potential

**Hui Li[1#], Jie Xu[1#], Zhong-Shan Shen[1#], Guang-Ming Wang[1#], Mingxi Tang[2], Xiang-Rong Du[1], Yan-Tian Lv[1], Jing-Jing Wang[1], Fei-Fei Zhang[1], Zhi Qi[3], Zhe Zhang[1], Masahiro Sokabe[4,5,6*] Qiong-Yao Tang[1,4*]**

**Supporting Information** (Fig. S1-Fig. S5)

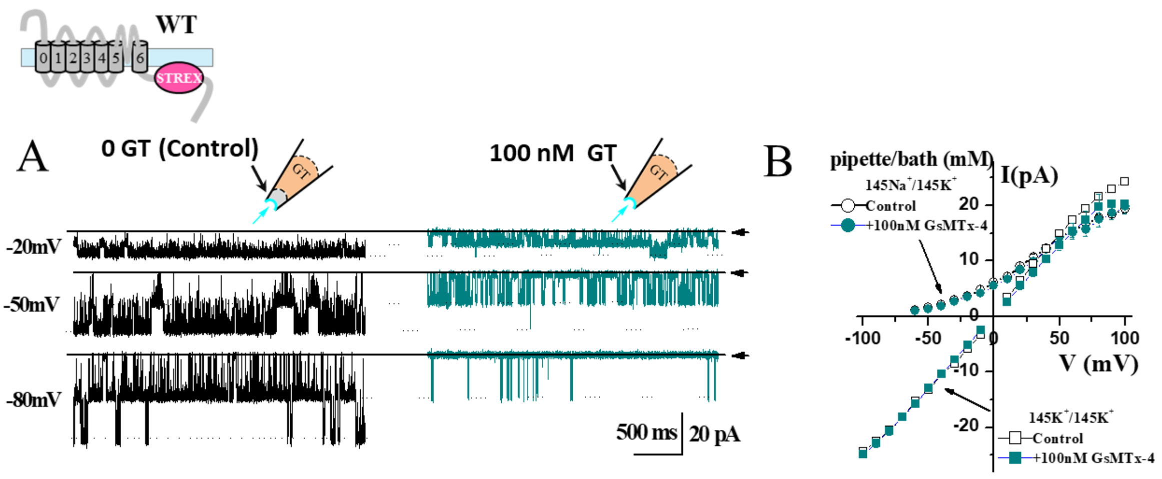


**Fig. S1. GsMTx4 inhibits stretch-activated BK channel without changing single-channel conductance**. (A) Single-channel current comparisons for SAKca channel between the conditions in the absence (left) and in the presence of 100 nM GsMTx4 applied in the pipette (see Method). The intracellular $Ca^{2+}$ (in the bath) used was 1 mM. Current traces were obtained from the same patches 30 min later from the onset of backfilling (see Methods). (B) *I-V* relationships comparisons of SAKcaC in the absence (control: open symbols) and presence (filled symbols) of 100 nM GsMTx4 in the pipette (see methods) with symmetrical $145K^+$ (squares) or 145 $Na^+$ (in pipette) /$145K^+$ (in the bath) (circles). Currents were recorded with 1mM $[Ca^{2+}]_i$ in the bath from chick ventricular myocytes. Data points in individual conditions represent averages of 4-16 patches. SAKca channels were recorded from chick ventricular myocytes

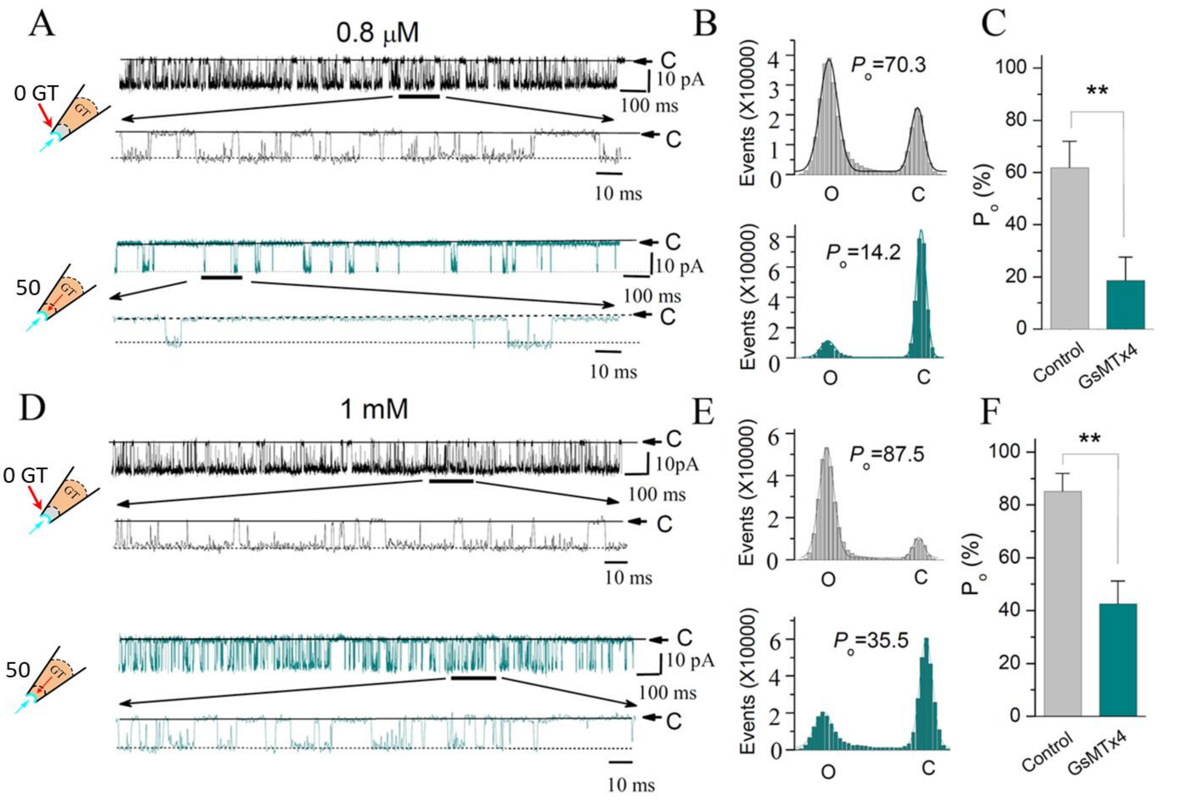


**Fig. S2. GsMTx4 inhibits SAKcaC in the presence of 0.8 μM $[Ca^{2+}]_i$.** For comparison, the same concentration of GsMTx4 effect in the presence of 1 mM $[Ca^{2+}]_i$ is presented in D-F.

(A) Typical single-channel current traces showing the inhibitory effect of 50 nM GsMTx4 on SAKcaC at –40 mV. Traces were obtained at the time points of 2 min (upper) and 25min (lower) following the onset of back-filling (See methods). The cartoons on the left show that GsMTx4 was applied from the pipette before (upper) and after (lower) 50 nM GsMTx4 completely reached the extracellular site of cell membrane. The intracellular $Ca^{2+}$ (in the bath) used was 0.8 μM.
(B) Total amplitude histogram events of channels open (O) and closed (C) states corresponding to A were fitted by Gaussian functions. $P_o$ values were 70.3 (upper) and 14.2 (lower), respectively.
(C) The summarized $P_o$ for control (left) and 50 nM GsMTx4 effect (right) on SAKcaC 25 min later following the onset of back-filling, the intracellular $Ca^{2+}$ (in the bath) used was 0.8 μM (n = 5).
(D) The same as in A, but the intracellular $Ca^{2+}$ (in the bath) used was 1mM.
(E) Total amplitude histogram events of channels open (O) and closed (C) states corresponding to D were fitted by Gaussian functions. $P_o$ values were 87.5 (upper) and 35.5 (lower), respectively.
(F) The summarized $P_o$ for control (left) and the effect of 50 nM GsMTx4 on SAKcaC (right) 25 min later following the onset of back-filling (n = 8), the intracellular $Ca^{2+}$ used (in the bath) was 1mM.

SAKcaCs were recorded from cultured chick ventricular myocytes

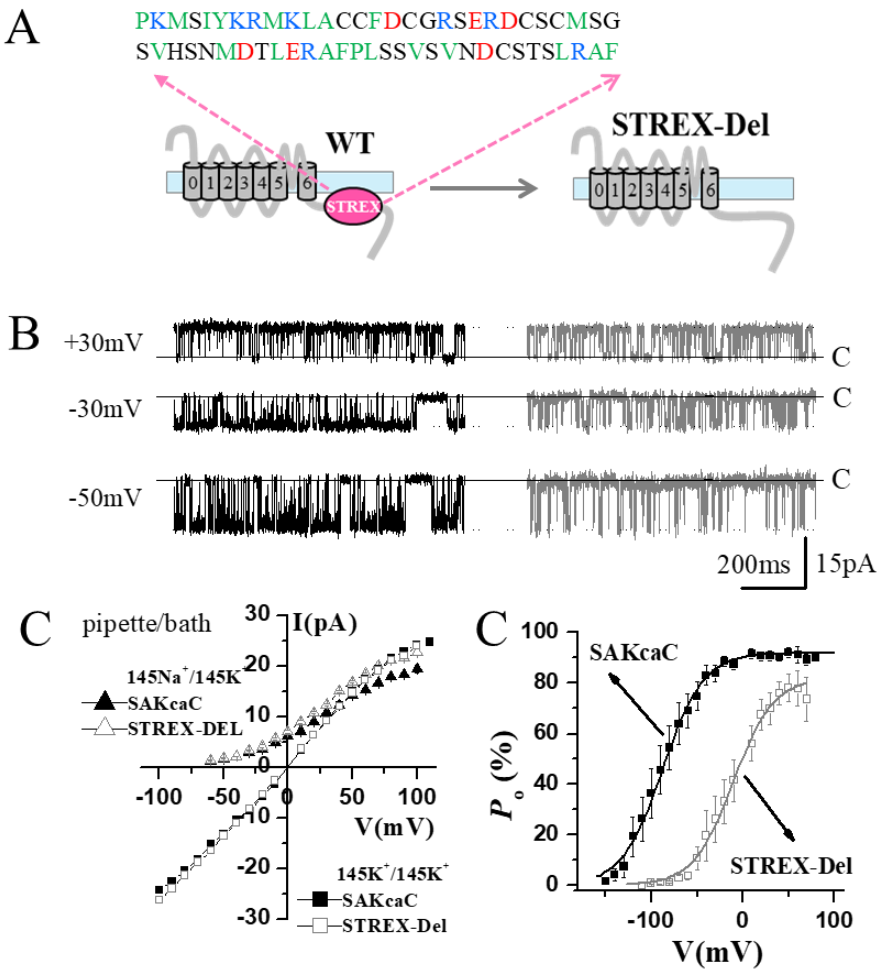


**Fig. S3. Deletion of the STREX-exon in the C-terminal (STREC-Del) decreased SAKcaC channel activity without changes in single-channel conductance or ion selectivity.**

(A) Sequence alignment for the STREX-exon located in the C-terminus of SAKcaC. The hydrophobic residues are highlighted by green and the positively charged residues are shown in blue (Arg, Lys).

(B) Single-channel current comparisons between SAKcaC (left) and STREX-Del (right). Membrane potentials (MPs) were held at + 30, -30 and -50 mV as indicated. Symbol C indicates the closed level of channel.

(C) *I*-*V* relationship comparisons between SAKcaC and STREC-Del channels. *I*-*V* relationships were measured with symmetrical $145K^+$ for SAKcaC (■ n>15) and STREC-Del (●, n>4) channels, or 145 $Na^+$ in the pipette and $145K^+$ in the bath for SAKcaC (□, n >6) and STREC-Del channel (○, n>5), respectively.

(D) $P_o$-$V$ relationship comparisons between SAKcaC (□) and STREX-Del (○) channels. The data were fitted with the standard Boltzmann function, $P_o = P_{o,\,max} / \{1 + \exp[(-(V_m - V_{1/2}) / K)]\}$, where $V_{1/2}$ represents the voltage at half-maximum $P_o$, and $K$ represents the slope factor. The $V_{1/2}$ and $K^{-1}$ obtained are: -88.6 ± 0.8 mV and 23.7 ± 1.3 for SAKcaC (n = 8), and -12.4 ± 1.6 mV and 23.1 ± 1.2 for STREX-De; channel (n>4).

Intracellular $[Ca^{2+}]_i$ concentrations used were 1 mM. SAKcaCs and STREX-Del mutation channels were recorded from cultured chick ventricular myocytes.

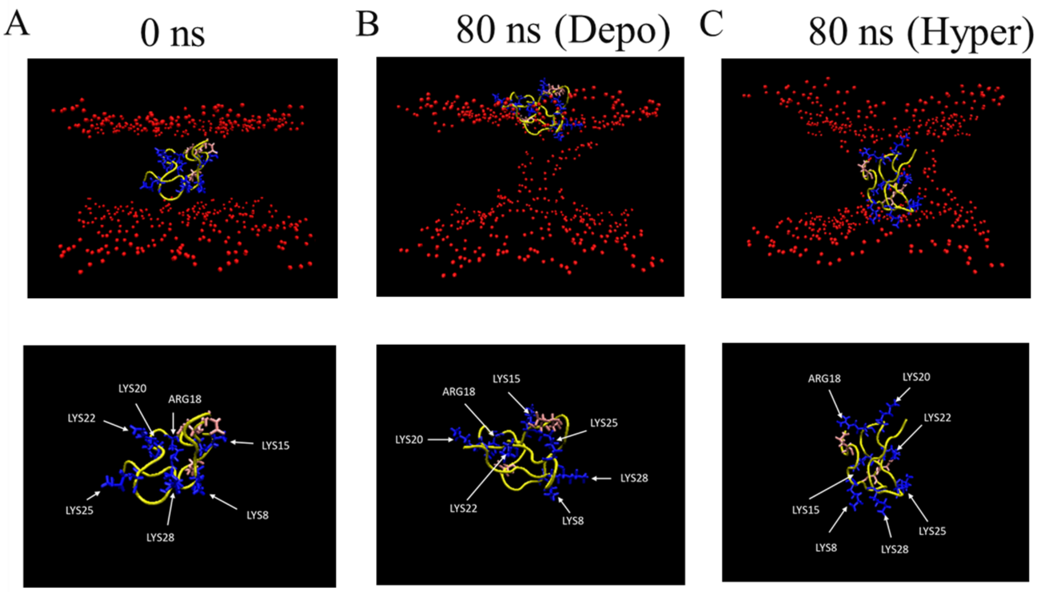


**Fig. S4. GsMTx4 in the lipid bilayer interacts with the carbonyl oxygen atoms under membrane depolarization (Depo) *vs.* hyperpolarization (Hyper) states.**

(A) Snapshot taken at the initial position of MD simulation (0 nS ). GsMTx4 com (the center of GsMTx4) was initially put at the location of ~0.5nm above the center of DPPC bilayer (See Fig. 10 insert). For clarity, only oxygen atoms (red spheres) contained in the lipid are shown. The panel in the below shows the detailed feature of GsMTx4 that was randomly put within the lipid bilayer corresponding to the orientation above.

(B) Snapshot taken at 80 nS under the condition of membrane depolarization (Depo). The panel in the below shows the detailed feature of GsMTx4 corresponding to the orientation above. The positively charged residues (blue) interacted with the carbonyl oxygen atoms at the outer leaflet lipid are indicated.

(C) Snapshot taken at 80 nS under the condition of membrane hyperpolarization (Hyper). The positively charged residues interacted with the monolayer of lipid bilayer are split into two groups: those of R18, K-20, K-22 are interacting with the carbonyl oxygen atoms in the outer leaflet lipid, and those of K-8, K-15, K-25, K28 are interacting with that in the inner leaflet lipid.

The peptide backbone is shown as a yellow tube. Positively charged residues (Arg, Lys) are shown in blue, and negatively charged residues (Asp, Glu) are in pink.

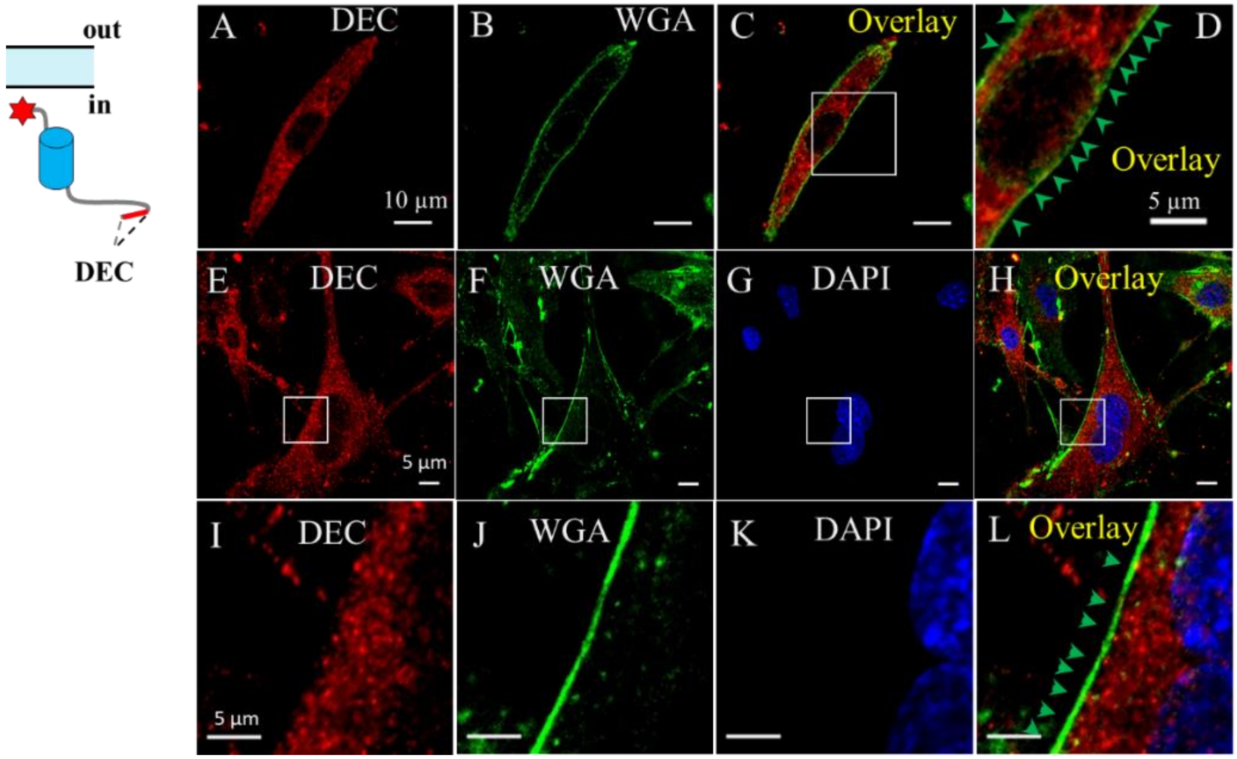


Fig. S5. BK variants containing DEC exon do not contribute to the cardiac myocyte membrane isolated from newborn mice. (A-B) Ventricular myocytes loaded with plasma membrane marker (WGA), fixed, permeabilized and labeled with anti-DEC Ab, a poly-cloned antibody against DEC at the C-terminal end of BK α-subunit. (C) Overlay of A, B. (D) Amplification of squared region in C. (E-G) Ventricular myocytes loaded with plasma membrane marker (WGA), fixed, permeabilized and labeled with DAPI and anti-DEC Ab. (H) Overlay of E, F and G. (I-L) Amplification of squared region in E-H, respectively. The Green arrows in D and L highlight the plasma membrane marker (WGA). Thus, the Red signals in D and L (the merge) represent BK variants containing DEC exon in BK C-terminus (e.g. BK-DEC and/or BK-STREX-DEC), are not located on the surface of ventricular myocyte. Cartoon in the left show BK variant with the tag at the N terminus. The ventricular myocytes were isolated from new-born mice.
